# 1-[1-(4-Bromo­phen­yl)ethyl­idene]-4-(2,4-dimeth­oxy­phen­yl)thio­semicarbazide

**DOI:** 10.1107/S1600536810022622

**Published:** 2010-06-18

**Authors:** Muhammad Yaqub, Humayun Pervez, Nadia Arif, M. Nawaz Tahir, Mazhar Hussain

**Affiliations:** a Department of Chemistry, Bahauddin Zakariya University, Multan 60800, Pakistan; b Department of Physics, University of Sargodha, Sargodha, Pakistan

## Abstract

In the title compound, C_17_H_18_BrN_3_O_2_S, the dihedral angle between the aromatic rings is 9.15 (17)°. A bifurcated intra­molecular N—H⋯(N,O) hydrogen bond generates two *S*(5) rings and a weak intra­molecular C—H⋯S inter­action completes an *S*(6) ring motif. In the crystal, inversion dimers linked by pairs of N—H⋯S hydrogen bonds generate *R*_2_^2^(8) loops and weak C—H⋯S and C—H⋯π inter­actions are also present.

## Related literature

For the pharmacological applications of thio­semicarbazones see: Beraldo & Gambino (2004 ▶); Pervez *et al.* (2008 ▶, 2010*a* ▶,*b* ▶). For related structures, see: Jian *et al.* (2005 ▶); Martínez *et al.* (2006 ▶). For graph-set notation, see: Bernstein *et al.* (1995 ▶).
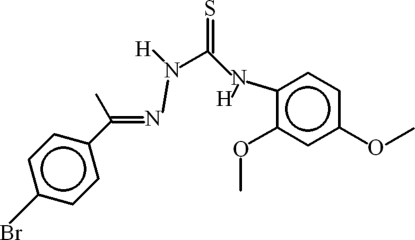


## Experimental

### 

#### Crystal data

C_17_H_18_BrN_3_O_2_S
*M* *_r_* = 408.31
Monoclinic, 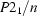

*a* = 5.8390 (2) Å
*b* = 30.3335 (11) Å
*c* = 9.9423 (4) Å
β = 94.910 (2)°
*V* = 1754.49 (11) Å^3^
*Z* = 4
Mo *K*α radiation
μ = 2.48 mm^−1^
*T* = 296 K
0.25 × 0.22 × 0.20 mm

#### Data collection

Bruker Kappa APEXII CCD diffractometer
Absorption correction: multi-scan (*SADABS*; Bruker, 2005 ▶) *T*_min_ = 0.642, *T*_max_ = 0.652
17115 measured reflections
4346 independent reflections
2654 reflections with *I* > 2σ(*I*)
*R*_int_ = 0.044

#### Refinement

*R*[*F* ^2^ > 2σ(*F* ^2^)] = 0.041
*wR*(*F* ^2^) = 0.100
*S* = 1.01
4346 reflections
220 parameters
H-atom parameters constrained
Δρ_max_ = 0.42 e Å^−3^
Δρ_min_ = −0.36 e Å^−3^

### 

Data collection: *APEX2* (Bruker, 2007 ▶); cell refinement: *SAINT* (Bruker, 2007 ▶); data reduction: *SAINT*; program(s) used to solve structure: *SHELXS97* (Sheldrick, 2008 ▶); program(s) used to refine structure: *SHELXL97* (Sheldrick, 2008 ▶); molecular graphics: *ORTEP-3* (Farrugia, 1997 ▶) and *PLATON* (Spek, 2009 ▶); software used to prepare material for publication: *WinGX* (Farrugia, 1999 ▶) and *PLATON*.

## Supplementary Material

Crystal structure: contains datablocks global, I. DOI: 10.1107/S1600536810022622/hb5499sup1.cif

Structure factors: contains datablocks I. DOI: 10.1107/S1600536810022622/hb5499Isup2.hkl

Additional supplementary materials:  crystallographic information; 3D view; checkCIF report

## Figures and Tables

**Table 1:** Hydrogen-bond geometry (Å, °) *Cg*1 is the centroid of C1–C6 ring.

*D*—H⋯*A*	*D*—H	H⋯*A*	*D*⋯*A*	*D*—H⋯*A*
N1—H1⋯O1	0.86	2.12	2.573 (3)	113
N1—H1⋯N3	0.86	2.05	2.538 (3)	115
N2—H2*A*⋯S1^i^	0.86	2.84	3.662 (2)	161
C2—H2⋯S1	0.93	2.58	3.248 (3)	129
C17—H17*A*⋯S1^ii^	0.96	2.86	3.774 (3)	161
C8—H8*A*⋯*Cg*1^iii^	0.96	2.98	3.860 (3)	153

Symmetry codes: (i) 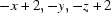; (ii) 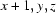; (iii) 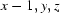.

**Fig. 1.**
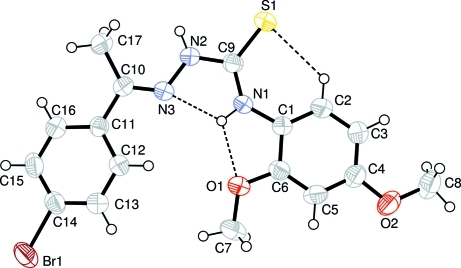
View of (I) with displacement ellipsoids drawn at the 50% probability level. The dotted lines indicate the intra-molecular H-bonds.

**Fig. 2.**
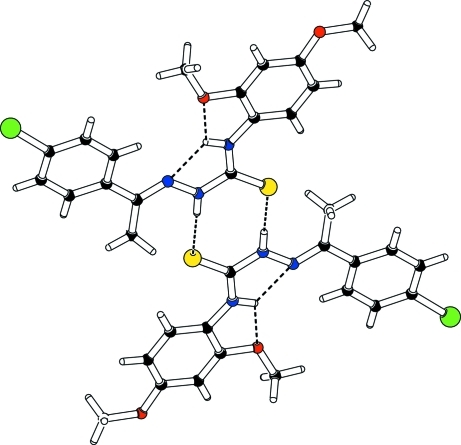
The partial packing of (I), which shows that molecules form dimers.

## supplementary crystallographic information

### Comment

Thiosemicarbazones have wide pharmacological properties (Beraldo & Gambino, 2004). Prompted by this, we recently reported the synthesis and medicinal importance of some isatins-thiosemicarbazones (Pervez *et al.*, 2008, 2010*a*,*b*). Now, we report the synthesis and crystal structure of the title compound (I), (Fig. I).

The crystal structure of (II) *i.e.* 4-fluoroacetophenone-*N*-propylthiosemicarbazone (Martinez *et al.*, 2006) and (III) *i.e.* 4-phenyl-1-(1-phenylethylidene)thiosemicarbazide (Jian, *et al.*, 2005) have been published. The title compound (I) is different from (II) and (III) due to attachment of substituants at the phenyl rings.

In (I), the phenyl ring A (C1–C6) of 2,4-dimethoxyanilino group, B (C11—C16) of 4-bromophenyl are planar with r. m. s. deviations of 0.0034 and 0.0036 Å, respectively. The thiosemicarbazone moiety C (N1—N3/C9/S1) is also planar with r. m. s. deviation of 0.0062 Å from its mean square plane. The dihedral angle between A/B, A/C and B/C is 9.15 (17), 2.07 (17) and 9.12 (16)°, respectively. Two S(5) ring motifs (Bernstein *et al.*, 1995) (Table 1, Fig. 1) are formed due to strong intramolecular H-bonding of N—H···N and N—H···O types. The weak interaction of C—H···S type completes an S(6) ring motif. The molecules are dimerized due to intermolecular interactions of N—H···S type and complete *R*_2_^2^(8) ring motif (Fig. 2). The dimers are interlinked through C—H···S interactions (Table 1). The C—H···π interaction (Table 1) also play role in stabilizing the molecules.

### Experimental

A solution of 4-(2,4-dimethoxyphenyl)thiosemicarbazide (0.15 g, 0.66 mole) in warm ethanol (20 ml) was added drop wise to the stirred solution of 4-bromoacetophenone (0.13 g, 0.66 mol) in warm ethanol (10 ml) containing 2–3 drops of acetic acid. The resultant mixture was then heated under reflux for 30 min. After cooling the reaction mixture to room temperature, the yellow solid was collected by suction filtration, washing with ethanol furnished the title compound in pure form (0.21 g, 95%), m.p. 502 K. Colourless prisms of (I) were grown in chloroform-petroleum ether (1:5) system at room temperature by diffusion method.

### Refinement

The H-atoms were positioned geometrically (N–H = 0.86 Å, C–H = 0.93–0.96 Å) and refined as riding with *U*_iso_(H) = *xU*_eq_(C, N), where *x* = 1.5 for methyl and *x* = 1.2 for all other H-atoms.

### Crystal data

**Table:**

C_17_H_18_BrN_3_O_2_S	*F*(000) = 832
*M**_r_* = 408.31	*D*_x_ = 1.546 Mg m^−^^3^
Monoclinic, *P*2_1_/*n*	Mo *K*α radiation, λ = 0.71073 Å
Hall symbol: -P 2yn	Cell parameters from 2654 reflections
*a* = 5.8390 (2) Å	θ = 2.2–28.3°
*b* = 30.3335 (11) Å	µ = 2.48 mm^−^^1^
*c* = 9.9423 (4) Å	*T* = 296 K
β = 94.910 (2)°	Prism, colorless
*V* = 1754.49 (11) Å^3^	0.25 × 0.22 × 0.20 mm
*Z* = 4	

### Data collection

**Table:**

Bruker Kappa APEXII CCD diffractometer	4346 independent reflections
Radiation source: fine-focus sealed tube	2654 reflections with *I* > 2σ(*I*)
graphite	*R*_int_ = 0.044
Detector resolution: 7.5 pixels mm^-1^	θ_max_ = 28.3°, θ_min_ = 2.2°
ω scans	*h* = −4→7
Absorption correction: multi-scan (*SADABS*; Bruker, 2005)	*k* = −40→40
*T*_min_ = 0.642, *T*_max_ = 0.652	*l* = −13→13
17115 measured reflections	

### Refinement

**Table:**

Refinement on *F*^2^	Primary atom site location: structure-invariant direct methods
Least-squares matrix: full	Secondary atom site location: difference Fourier map
*R*[*F*^2^ > 2σ(*F*^2^)] = 0.041	Hydrogen site location: inferred from neighbouring sites
*wR*(*F*^2^) = 0.100	H-atom parameters constrained
*S* = 1.01	*w* = 1/[σ^2^(*F*_o_^2^) + (0.0413*P*)^2^ + 0.4088*P*] where *P* = (*F*_o_^2^ + 2*F*_c_^2^)/3
4346 reflections	(Δ/σ)_max_ = 0.001
220 parameters	Δρ_max_ = 0.42 e Å^−^^3^
0 restraints	Δρ_min_ = −0.36 e Å^−^^3^

### Special details

**Table:**

Geometry. Bond distances, angles *etc*. have been calculated using the rounded fractional coordinates. All su's are estimated from the variances of the (full) variance-covariance matrix. The cell e.s.d.'s are taken into account in the estimation of distances, angles and torsion angles
Refinement. Refinement of *F*^2^ against ALL reflections. The weighted *R*-factor *wR* and goodness of fit *S* are based on *F*^2^, conventional *R*-factors *R* are based on *F*, with *F* set to zero for negative *F*^2^. The threshold expression of *F*^2^ > σ(*F*^2^) is used only for calculating *R*-factors(gt) *etc*. and is not relevant to the choice of reflections for refinement. *R*-factors based on *F*^2^ are statistically about twice as large as those based on *F*, and *R*- factors based on ALL data will be even larger.

### Fractional atomic coordinates and isotropic or equivalent isotropic displacement parameters (Å^2^)

**Table:**

	*x*	*y*	*z*	*U*_iso_*/*U*_eq_	
Br1	1.68144 (7)	0.28876 (1)	0.66065 (3)	0.0846 (1)	
S1	0.69373 (12)	0.00989 (2)	0.87217 (7)	0.0577 (2)	
O1	0.7242 (3)	0.14610 (6)	0.55058 (18)	0.0598 (7)	
O2	0.0390 (3)	0.09126 (7)	0.3147 (2)	0.0710 (8)	
N1	0.7530 (3)	0.08160 (7)	0.7192 (2)	0.0504 (7)	
N2	1.0174 (3)	0.06989 (7)	0.8946 (2)	0.0490 (7)	
N3	1.1192 (3)	0.10729 (7)	0.8520 (2)	0.0465 (7)	
C1	0.5629 (4)	0.08091 (8)	0.6222 (2)	0.0453 (8)	
C2	0.3956 (5)	0.04927 (10)	0.6085 (3)	0.0684 (11)	
C3	0.2178 (5)	0.05173 (10)	0.5075 (3)	0.0707 (11)	
C4	0.2059 (4)	0.08609 (9)	0.4186 (3)	0.0536 (9)	
C5	0.3721 (4)	0.11870 (9)	0.4316 (3)	0.0519 (9)	
C6	0.5493 (4)	0.11606 (8)	0.5315 (2)	0.0449 (8)	
C7	0.7459 (5)	0.17845 (10)	0.4497 (3)	0.0718 (11)	
C8	−0.1278 (5)	0.05715 (11)	0.2945 (3)	0.0714 (11)	
C9	0.8214 (4)	0.05526 (8)	0.8225 (2)	0.0426 (8)	
C10	1.2991 (4)	0.12299 (8)	0.9180 (2)	0.0413 (8)	
C11	1.3922 (4)	0.16334 (8)	0.8596 (2)	0.0397 (7)	
C12	1.2659 (4)	0.18592 (9)	0.7571 (3)	0.0522 (9)	
C13	1.3493 (5)	0.22333 (9)	0.7001 (3)	0.0585 (10)	
C14	1.5632 (4)	0.23844 (9)	0.7440 (3)	0.0511 (9)	
C15	1.6935 (5)	0.21732 (9)	0.8453 (3)	0.0560 (10)	
C16	1.6074 (4)	0.17997 (9)	0.9025 (3)	0.0508 (9)	
C17	1.4082 (5)	0.10312 (9)	1.0456 (3)	0.0565 (9)	
H1	0.84389	0.10350	0.71009	0.0605*	
H2	0.40157	0.02564	0.66831	0.0820*	
H2A	1.07456	0.05588	0.96481	0.0587*	
H3	0.10583	0.02985	0.50022	0.0849*	
H5	0.36375	0.14251	0.37241	0.0623*	
H7A	0.61321	0.19727	0.44397	0.1075*	
H7B	0.75783	0.16426	0.36429	0.1075*	
H7C	0.88133	0.19576	0.47252	0.1075*	
H8A	−0.21919	0.05609	0.37027	0.1075*	
H8B	−0.05148	0.02938	0.28602	0.1075*	
H8C	−0.22514	0.06296	0.21373	0.1075*	
H12	1.12106	0.17552	0.72613	0.0626*	
H13	1.26085	0.23820	0.63226	0.0701*	
H15	1.83820	0.22799	0.87536	0.0672*	
H16	1.69579	0.16564	0.97145	0.0610*	
H17A	1.48971	0.07685	1.02462	0.0848*	
H17B	1.29134	0.09589	1.10418	0.0848*	
H17C	1.51361	0.12388	1.08973	0.0848*	

### Atomic displacement parameters (Å^2^)

**Table:**

	*U*^11^	*U*^22^	*U*^33^	*U*^12^	*U*^13^	*U*^23^
Br1	0.1088 (3)	0.0700 (2)	0.0736 (2)	−0.0456 (2)	0.0001 (2)	0.0106 (2)
S1	0.0585 (4)	0.0489 (4)	0.0642 (4)	−0.0114 (3)	−0.0030 (3)	0.0194 (3)
O1	0.0677 (12)	0.0508 (11)	0.0587 (12)	−0.0148 (9)	−0.0080 (9)	0.0172 (9)
O2	0.0668 (12)	0.0697 (14)	0.0712 (13)	0.0015 (11)	−0.0242 (10)	0.0076 (11)
N1	0.0543 (12)	0.0453 (12)	0.0496 (12)	−0.0150 (10)	−0.0074 (10)	0.0128 (10)
N2	0.0522 (12)	0.0458 (12)	0.0470 (12)	−0.0095 (10)	−0.0066 (10)	0.0129 (10)
N3	0.0493 (12)	0.0431 (12)	0.0464 (12)	−0.0070 (9)	−0.0005 (10)	0.0087 (9)
C1	0.0483 (14)	0.0439 (14)	0.0429 (13)	−0.0044 (11)	−0.0008 (11)	0.0061 (11)
C2	0.077 (2)	0.0571 (18)	0.0670 (19)	−0.0215 (15)	−0.0181 (16)	0.0215 (15)
C3	0.0721 (19)	0.063 (2)	0.072 (2)	−0.0253 (16)	−0.0223 (16)	0.0155 (16)
C4	0.0529 (16)	0.0520 (16)	0.0540 (16)	0.0031 (12)	−0.0056 (13)	−0.0032 (13)
C5	0.0644 (17)	0.0443 (14)	0.0462 (15)	0.0062 (12)	−0.0006 (13)	0.0072 (11)
C6	0.0517 (15)	0.0390 (13)	0.0442 (14)	−0.0022 (11)	0.0051 (12)	0.0012 (10)
C7	0.090 (2)	0.0543 (19)	0.070 (2)	−0.0172 (16)	0.0010 (17)	0.0197 (15)
C8	0.0591 (18)	0.076 (2)	0.075 (2)	0.0018 (16)	−0.0180 (15)	−0.0104 (17)
C9	0.0461 (14)	0.0407 (13)	0.0412 (13)	−0.0013 (10)	0.0043 (11)	0.0027 (10)
C10	0.0435 (14)	0.0416 (13)	0.0384 (13)	0.0037 (10)	0.0018 (11)	−0.0006 (10)
C11	0.0417 (13)	0.0388 (13)	0.0384 (12)	−0.0005 (10)	0.0025 (10)	−0.0044 (10)
C12	0.0475 (15)	0.0506 (15)	0.0572 (16)	−0.0094 (12)	−0.0030 (12)	0.0095 (13)
C13	0.0617 (18)	0.0552 (17)	0.0567 (17)	−0.0082 (13)	−0.0053 (14)	0.0109 (13)
C14	0.0603 (16)	0.0469 (15)	0.0465 (15)	−0.0135 (12)	0.0075 (13)	−0.0047 (12)
C15	0.0539 (16)	0.0591 (18)	0.0544 (16)	−0.0181 (13)	0.0004 (13)	−0.0090 (13)
C16	0.0507 (15)	0.0525 (16)	0.0480 (14)	−0.0024 (12)	−0.0029 (12)	0.0005 (12)
C17	0.0650 (17)	0.0505 (16)	0.0515 (16)	−0.0036 (13)	−0.0099 (13)	0.0063 (12)

### Geometric parameters (Å, °)

**Table:**

Br1—C14	1.895 (3)	C11—C12	1.387 (4)
S1—C9	1.660 (2)	C12—C13	1.376 (4)
O1—C6	1.370 (3)	C13—C14	1.366 (4)
O1—C7	1.417 (3)	C14—C15	1.368 (4)
O2—C4	1.367 (3)	C15—C16	1.382 (4)
O2—C8	1.423 (4)	C2—H2	0.9300
N1—C1	1.407 (3)	C3—H3	0.9300
N1—C9	1.335 (3)	C5—H5	0.9300
N2—N3	1.365 (3)	C7—H7A	0.9600
N2—C9	1.371 (3)	C7—H7B	0.9600
N3—C10	1.282 (3)	C7—H7C	0.9600
N1—H1	0.8600	C8—H8A	0.9600
N2—H2A	0.8600	C8—H8B	0.9600
C1—C2	1.368 (4)	C8—H8C	0.9600
C1—C6	1.394 (3)	C12—H12	0.9300
C2—C3	1.383 (4)	C13—H13	0.9300
C3—C4	1.365 (4)	C15—H15	0.9300
C4—C5	1.384 (4)	C16—H16	0.9300
C5—C6	1.374 (3)	C17—H17A	0.9600
C10—C11	1.478 (3)	C17—H17B	0.9600
C10—C17	1.497 (4)	C17—H17C	0.9600
C11—C16	1.387 (3)		
			
C6—O1—C7	118.27 (19)	C14—C15—C16	119.2 (3)
C4—O2—C8	117.3 (2)	C11—C16—C15	121.6 (3)
C1—N1—C9	133.2 (2)	C1—C2—H2	119.00
N3—N2—C9	118.23 (19)	C3—C2—H2	119.00
N2—N3—C10	120.6 (2)	C2—C3—H3	120.00
C9—N1—H1	113.00	C4—C3—H3	120.00
C1—N1—H1	113.00	C4—C5—H5	120.00
N3—N2—H2A	121.00	C6—C5—H5	120.00
C9—N2—H2A	121.00	O1—C7—H7A	109.00
C2—C1—C6	118.1 (2)	O1—C7—H7B	109.00
N1—C1—C6	115.5 (2)	O1—C7—H7C	109.00
N1—C1—C2	126.4 (2)	H7A—C7—H7B	109.00
C1—C2—C3	121.3 (3)	H7A—C7—H7C	109.00
C2—C3—C4	120.3 (3)	H7B—C7—H7C	109.00
O2—C4—C3	124.8 (2)	O2—C8—H8A	109.00
O2—C4—C5	115.9 (2)	O2—C8—H8B	109.00
C3—C4—C5	119.3 (3)	O2—C8—H8C	109.00
C4—C5—C6	120.3 (3)	H8A—C8—H8B	109.00
O1—C6—C5	124.6 (2)	H8A—C8—H8C	109.00
C1—C6—C5	120.7 (2)	H8B—C8—H8C	109.00
O1—C6—C1	114.75 (19)	C11—C12—H12	119.00
S1—C9—N1	127.77 (18)	C13—C12—H12	119.00
S1—C9—N2	119.25 (17)	C12—C13—H13	120.00
N1—C9—N2	112.9 (2)	C14—C13—H13	120.00
C11—C10—C17	121.5 (2)	C14—C15—H15	120.00
N3—C10—C11	114.80 (19)	C16—C15—H15	120.00
N3—C10—C17	123.7 (2)	C11—C16—H16	119.00
C10—C11—C16	122.3 (2)	C15—C16—H16	119.00
C12—C11—C16	117.2 (2)	C10—C17—H17A	109.00
C10—C11—C12	120.5 (2)	C10—C17—H17B	109.00
C11—C12—C13	121.7 (2)	C10—C17—H17C	109.00
C12—C13—C14	119.4 (3)	H17A—C17—H17B	109.00
Br1—C14—C13	119.2 (2)	H17A—C17—H17C	109.00
C13—C14—C15	120.9 (3)	H17B—C17—H17C	110.00
Br1—C14—C15	119.90 (19)		
			
C7—O1—C6—C1	169.2 (2)	C2—C3—C4—O2	179.3 (3)
C7—O1—C6—C5	−10.1 (3)	C2—C3—C4—C5	−0.8 (4)
C8—O2—C4—C3	−3.1 (4)	O2—C4—C5—C6	−178.9 (2)
C8—O2—C4—C5	176.9 (2)	C3—C4—C5—C6	1.1 (4)
C9—N1—C1—C2	−2.8 (4)	C4—C5—C6—O1	178.5 (2)
C9—N1—C1—C6	178.0 (2)	C4—C5—C6—C1	−0.8 (4)
C1—N1—C9—S1	0.2 (4)	N3—C10—C11—C12	11.5 (3)
C1—N1—C9—N2	−177.7 (2)	N3—C10—C11—C16	−167.7 (2)
C9—N2—N3—C10	178.1 (2)	C17—C10—C11—C12	−167.6 (2)
N3—N2—C9—S1	−179.91 (16)	C17—C10—C11—C16	13.1 (4)
N3—N2—C9—N1	−1.8 (3)	C10—C11—C12—C13	−179.4 (2)
N2—N3—C10—C11	179.44 (19)	C16—C11—C12—C13	−0.1 (4)
N2—N3—C10—C17	−1.4 (3)	C10—C11—C16—C15	178.9 (2)
N1—C1—C2—C3	−179.0 (3)	C12—C11—C16—C15	−0.3 (4)
C6—C1—C2—C3	0.2 (4)	C11—C12—C13—C14	0.9 (4)
N1—C1—C6—O1	0.1 (3)	C12—C13—C14—Br1	177.8 (2)
N1—C1—C6—C5	179.5 (2)	C12—C13—C14—C15	−1.2 (4)
C2—C1—C6—O1	−179.3 (2)	Br1—C14—C15—C16	−178.2 (2)
C2—C1—C6—C5	0.2 (4)	C13—C14—C15—C16	0.7 (4)
C1—C2—C3—C4	0.1 (4)	C14—C15—C16—C11	0.1 (4)

### Hydrogen-bond geometry (Å, °)

**Table:**

Cg1 is the centroid of C1–C6 ring.

**Table:**

*D*—H···*A*	*D*—H	H···*A*	*D*···*A*	*D*—H···*A*
N1—H1···O1	0.86	2.12	2.573 (3)	113
N1—H1···N3	0.86	2.05	2.538 (3)	115
N2—H2A···S1^i^	0.86	2.84	3.662 (2)	161
C2—H2···S1	0.93	2.58	3.248 (3)	129
C17—H17A···S1^ii^	0.96	2.86	3.774 (3)	161
C8—H8A···Cg1^iii^	0.96	2.98	3.860 (3)	153

Symmetry codes: (i) −*x*+2, −*y*, −*z*+2; (ii) *x*+1, *y*, *z*; (iii) *x*−1, *y*, *z*.
